# Literary

**Published:** 1872-10

**Authors:** 


LITERARY.—Messrs. Lindsay and Blakiston have issued a new catalogue of Medical Works published by them. They have added to their list of works many new and valuable text books. Any of our readers desiring a copy of the catalogue can obtain one by sending their address to the publishers No. 25 South Sixth Street, Philadelphia, Pa. The high standing of this house will be itself a recommendation to any work published by them.

Messrs. Locke & Jones, Toledo, Ohio, announce that they will issue, November 1st, the initial number of a new literary magazine to be called Locke's Dollar Monthly, with Mr. D. R. Locke, (Nasby) as editor. The well known name of Nasby as editor can not fail to attract subscribers. The price of the magazine is one dollar a year. Those subscribing before Nov. 15th will receive the magazine for two years for one dollar.

